# Vastus medialis oblique and vastus lateralis activity during a double-leg semisquat with or without hip adduction in patients with patellofemoral pain syndrome

**DOI:** 10.1186/s12891-015-0736-6

**Published:** 2015-10-12

**Authors:** Ping Miao, Yi Xu, Cuihuan Pan, Hao Liu, Chuhuai Wang

**Affiliations:** 1 Department of Rehabilitation Medicine, the Second Affiliated Hospital of Guangzhou Medical University, Guangzhou, China; 2 Department of Rehabilitation Medicine, the First Affiliated Hospital of Sun Yat-Sen University, Guangzhou, China; 3 Department of Physical Therapy, University of North Texas, Health Science Center, Fort Worth, Texas, USA

**Keywords:** Patellofemoral pain syndrome, Vastus medialis obliquus, Hip adduction, Surface electromyography

## Abstract

**Background:**

The purpose was to investigate the effect of double-leg semisquat with hip adduction on the activation of vastus medialis oblique (VMO) and vastus lateralis (VL) in patients with patellofemoral pain syndrome (PFPS).

**Methods:**

Thirty patients with PFPS were designated to the study group, while 30 healthy matched subjects were enrolled in the control group. The activation of VL and VMO was recorded with surface electromyography (EMG) during double-leg semisquat (DS) and double-leg semisquat with hip adduction (DS-HA). The time domain and frequency domain indexes of the electromyography data were collected for analysis.

**Results:**

In the study group, the time domain indexes (RMS, IEMG) and frequency domain index (MPF) of VL were significant higher than VMO in the test of DS (*P* < 0.05); and the time domain of VMO was significantly higher in the test of DS-HA when compared to DS (*P* < 0.05) while there was no difference in the activation of VL.

**Conclusions:**

In the study group, an increase in activity of the VMO was observed through the surface EMG signal in the double-leg semisquat exercise with hip adduction compared to the exercise without hip adduction. This finding indicates that VMO activation can be more selectively obtained through the exercise with hip adduction which can help balance the VL and VMO.

## Background

Patellofemoral pain syndrome (PFPS) can be defined as retropatellar or peripatellar pain resulting from physical and biomechanical changes in the patellofemoral joint. The pain is most prominent when ascending or descending stairs, squatting, or prolonged sitting with the knees flexed [1]. This is the most common diagnosis [1] in outpatients complaining of knee pain. PFPS has an incidence between 15 and 25 % [2], accounting for 25 % [3] of sports-related knee injuries. However, consensus is still lacking regarding the etiology, classification, diagnosis and treatment of the syndrome. The cause of patellofemoral pain has been reported to be multifactorial [2]. Various risk factors for PFPS have been suggested [1], including overuse, trauma, anatomic factors, muscle dysfunction, and lower extremity malalignment. Quadriceps insufficiency or atrophy, especially the unbalanced action of the quadriceps components, has been frequently considered the most important biochemical pathogenesis of PFPS though there is little objective evidence [2, 4].

Vastus lateralis (VL) is generally considered to cause the patella to move laterally, and vastus medialis, especially vastus medialis oblique (VMO), pulls the patella medially. The fibers of the VMO are angled 50–55° (with respect to the shaft of the femur), which is almost horizontal, and therefore the VMO is considered the primary medial stabilizer of the patella and has little function in knee extension. The VMO and VL align the patella within the trochlear groove as the knee moves through flexion and extension. This view has been supported by research based on the anatomy, imaging, and/or electrophysiology [5–7]. Therefore, imbalances in activation of these muscles can cause patellar malalignment. Muscular weakness of the VMO could lead to patellar lateral tracking and abnormal changes in patellofemoral joint pressure, producing knee joint instability, subsequently followed by pain, dysfunction and pathological changes of the patella cartilage [8]. Some research [4, 9–11] confirms that patients with patellofemoral problems exhibit atrophy of the VMO as indicated by magnetic resonance imaging (MRI), computed tomography (CT) or ultrasonography which may indicate an association between VMO weakness and patellar malalignment. Therefore, strengthening the VMO is commonly recommended as the focus of rehabilitation for patients with PFPS [8].

However, there is limited evidence regarding the efficacy of exercise therapy in treating PFPS, especially with respect to functional improvement of the VMO. The double-leg semisquat with hip adduction is a common exercises used in the rehabilitation of PFPS. Clinicians often attempt to facilitate VMO activity by instructing patients to squeeze a ball between their knees during squatting exercises, but this conventional clinical practice of VMO strengthening for PFPS has been challenged due to lack of objective evidence [12]. There has not been sufficient evidence concerning the effect of hip adduction on VMO strengthening in the past. VMO and VL activity during static and dynamic squat with isometric hip adduction has been investigated in healthy subjects [13–18]. Some research suggests that hip adduction might be useful in promoting a greater VMO/VL ratio [13–15], while other research apparently does not agree [16–18]. With respect to patients with PFPS, currently only two surface EMG studies have been performed and they fail to reach a consensus. One reported that hip adduction performed with a squat promoted a greater balance between the medial and lateral portions of the quadriceps femoris muscle but there was no preferential VMO muscle activation [19]. The second study suggested that hip adduction did not result in greater recruitment of the VMO muscle when compared with the VL muscle [20]. All subjects enrolled in these two studies were female which may impact the results because of the potential biomechanical differences between genders, additionally the sample size was too small (8 ~ 22 subjects) to draw convincing conclusions. In most of the research [13, 15–17, 19] mentioned above, only one time domain index (Root Mean Square – RMS) was used for analysis, which resulted in difficulty determining the overall recruitment and fatigue of the muscles. Also, some studies [13, 16, 18] failed to standardize the hip adduction force and had limitations in standardized posture selection. Therefore, further research is needed, and consideration should be given to methodological aspects of the study design.

With consideration of the above mentioned, the purpose of this study was to investigate the effect of double-leg semisquat with hip adduction on VL and VMO activation in patients with PFPS and healthy subjects by comparing the surface EMG characteristics and both the time domain and the frequency domain indexes of VMO and VL. It will help us to further understand the biomechanics of PFPS and provide guidance and an experimental basis for clinical evaluation and intervention for patients with PFPS.

## Methods

### Subjects

This is a cross-sectional study. Thirty patients with PFPS were chosen as the study group (SG), while 30 healthy subjects were enrolled as the control group (CG) and were matched with the study group for age, gender, height, and weight. The diagnosis of PFPS and eligibility for the study were established on the basis of clinical evaluation by an experienced physiatrist of the principle investigator’s (PI’s) institution. The subjects in the CG were recruited from the staff and students from PI’s institution. All healthy subjects had no current or previous record of knee pain, trauma, surgery, or other lower extremity disease. The sample size was determined by power analysis using preliminary data obtained in our laboratory. Subjects were recruited in the First Affiliated Hospital of Sun Yat-Sen University, and all experimental procedures and measurements were conducted in the physical therapy research lab of the hospital from 2011 to 2013. The study was approved by the ethics committee of the First Affiliated Hospital of Sun Yat-Sen University. All the subjects signed consent forms.

***The diagnostic criteria for PFPS*** [3, 21] were (1) anterior or retropatellar knee pain provoked by at least 2 of the following activities: prolonged sitting with flexed knees, squatting, ascending, stair climbing, kneeling, running, and jumping; and (2) exhibition of 2 or more of the following clinical criteria on assessment: pain with direct compression of the patella against the femoral condyles with the knee in full extension, tenderness on palpation of the posterior edge at the medial and/or lateral border of the patella, pain with resisted knee extension, and pain with direct compression of the patella against the femur during isometric quadriceps contraction with the knee in slight flexion.

***The inclusion criteria for SG*** included (1) age between 20 and 40 years (to reduce the likelihood of osteoarthritic changes in the patellofemoral joint); (2) the presence of patellofemoral pain for at least 3 months; and (3) scored less than six on the Visual Analogue Scale (VAS) during the peak of the pain. The patients did not receive any treatment for PFPS before entering the study.

***The exclusion criteria for SG*** consisted of (1) history or evidence of other knee disorders like patellar tendon pathology, ligament injury, bursitis, Osgood-Schlatter disease, meniscal lesion, or osteoarthritis; (2) lower limb surgery or trauma within one year; and (3) history of patellar dislocation or subluxation.

Twenty of a total of 30 PFPS patients in the SG reported bilateral knee pain. The leg that was more severely affected according to the patients’ complaints was used for the analysis. Among patients with unilateral PFPS, four had patellofemoral pain on the left and six on the right side. Within the CG, the leg that was used for assessment was randomly selected. All subjects in both groups were required to refrain from strenuous physical activity during the 24 h before the test.

### Instrumentation

The EMG activity of the VMO and VL was recorded with pregelled, round, Ag–AgCl surface electrodes (Shenfeng Medical Technologies, Shanghai, China) with a 10 mm contact area, and an inter-electrode distance of 20 mm. Signals were detected with a MegaWin ME3000 device (Mega Electronics Ltd, Kuopio, Finland) and displayed and analyzed by the Megawin PC software program (MegaWin software version 2.3, Mega Electronics Ltd, Kuopio, Finland). EMG data was amplified 2000 times and filtered between 20 and 500 Hz.

### Procedures

#### Double-leg semisquat (DS)

**Fig. 1:**
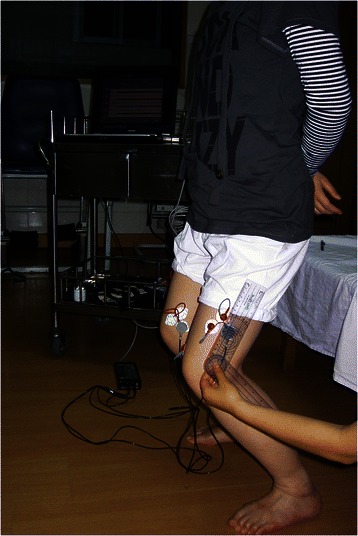
Double-leg semisquat (DS) at 60° knee flexion

Double-leg semisquat (DS) was performed with the knee flexed at 60° while maintaining the trunk in the upright position for one minute as shown in Fig. 1. Feet were shoulder-width apart and both feet were angled forward. A universal goniometer was used to monitor the angle of knee flexion in real time as shown in Fig. 1. The reason we adopted one minute for this study was based on our pilot study with five subjects. The pilot study showed that at 60° of knee flexion, patients with PFPS complained of knee pain and the quadriceps muscle would fatigue after a minimum of 70 to 90 s. So, for the purpose of safety, we selected 1 min for the testing time.

#### Double leg semisquat with hip adduction (DS-HA)

**Fig. 2:**
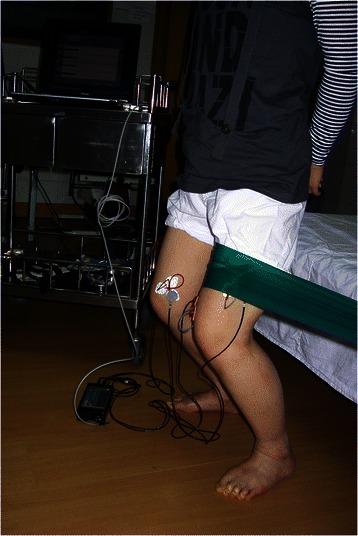
Double-leg semisquat with hip adduction (DS-HA) against a green thera-band

For the double leg semisquat exercise with hip adduction (DS-HA), subjects were instructed to perform DS with simultaneous hip adduction using a green Thera-Band® Resistance Band (Hygenic Corporation, Akron, US) which concurrently provided resistance to hip adduction as shown in Fig. 2. The band was stretched 2.5 times its resting length (measured by ruler) which produced a resistance of 9.6 lb at that elongation [22]. The subjects were required to maintain an upright posture instead of leaning towards one side, and were continuously encouraged with verbal commands when performing DS-HA.

#### Electrode placement

The detection electrodes were approximately placed parallel to the muscle fibers over the midline of the muscle belly, based on the positioning method suggested in previous studies [19, 21]. A reference line, from the anterosuperior iliac spine to the center of the patella, was drawn with a pen to help define the correct position of the surface EMG electrodes. The electrode for VMO was placed approximately 4 cm superior and medial to the superomedial border of the patella at a 50–55° angle to the reference line. The electrode for VL was placed 10 cm superior to the superolateral border of the patella at approximately 15–20° to the reference line. The inter-electrode distance was less than 3 cm. The reference electrode was positioned at the tibial tuberosity. To minimize the contact impedance, the skin was shaved and rubbed with abrasive gel and alcohol to carefully prepare it. Prior to the start of the experiments, there was a warm-up session which included 5-min of static stretching for the quadriceps, hamstring, and gastrocnemius muscles and a 5-min stationary bike exercise for the lower extremities to acclimate the subjects to the tests.

#### EMG data measurement and processing

The surface EMG activity of both VL and VMO were recorded for one-minute during the isometric contraction phase of the exercise when the subject performed DS or DS-HA. The order of the exercises was randomly assigned with a 10-min interval for the muscles to get fully rested to minimize the effects of muscle fatigue. Both the time domain indexes (Root Mean Square – RMS; Integrated EMG – IEMG), which reflect the level of physiological activity of motor units and overall muscle effort (contraction), and the frequency domain indexes (Median Frequency – MF, Mean Power Frequency – MPF), which reflect the muscle fatigue (motor unit firing rate and recruitment), were collected through the data extraction of Megawin PC software.

### Statistical analysis

The statistical analyses were performed using SPSS 15.0 for Windows (SPSS Inc., Chicago, USA). Descriptive statistics (mean and standard deviation) were used to describe the EMG activity for each muscle. An independent t-test was used to study the differences of the variables in the two groups at baseline. Comparison between the muscles and between the exercises was carried out within each group. Paired t tests were used to compare the EMG activity between the VMO and VL in order to determine if there were unbalanced actions of the quadriceps components between the different exercises and the different groups. Also, the EMG activity of VMO and VL between the two types of exercises was compared by paired t tests to identify whether hip adduction led to preferential VMO muscle activation. The significance level was set at *P* < 0.05. The consistency of the dependent variables over the trials was determined by using the intraclass correlation coefficient (ICC).

## Results

There were no significant differences between the two groups for the variables listed in Table 1, which indicated that the subjects in both groups were comparable (Table 1).

**Table 1:** Demographic data for study participants

	SG (*n* = 30)	CG (*n* = 30)	*P*
Age (year)	31.30 ± 6.87	28.70 ± 5.84	0.121
^*^Gender (male : female)	16:14	14:16	0.797
Height (cm)	165.23 ± 9.30	164.50 ± 8.46	0.750
Weight (kg)	60.60 ± 12.49	59.03 ± 10.74	0.604
Q-angle (degree)	14.38 ± 3.28	14.02 ± 3.38	0.137
Duration of symptoms (month)	60.86 ± 12.75	-	

^*^A chi-square test was used

The mean ICCs of the dependent variables were 0.93 ± 0.03, indicating that there were no significant differences in the variables among the trials. It is concluded that the results obtained were highly repeatable, allowing generalization of results. The power of this study was 0.63 and the effect size was found to be 0.88, which was sufficiently large enough to reveal any significant differences in this study.

All subjects completed the study. For the CG, the differences in the time and frequency domain indexes between VL and VMO were not significant in either DS or DS-HA test (*P* > 0.05). However, the SG presented higher MPF, RMS, IEMG of VL than VMO in the test of DS (*P* = 0.010, 0.007, and 0.013, respectively) and no such significant difference in the test of DS-HA (*P* > 0.05). Further comparison demonstrated there was no significant difference between DS and DS-HA test for the activity of VL or VMO within CG (*P* > 0.05) and VL within SG (*P* > 0.05). Still within SG, the greater RMS and IEMG of VMO were shown in DS-HA when compared with DS (*P* = 0.010 and 0.012, respectively) (Table 2).

**Table 2:** Mean and standard deviation, 95 % CI of EMG activity of the VMO and VL muscles

	VL				VMO			
	MF(Hz)	MPF(Hz)	RMS(μV)	IEMG(μVs)	MF(Hz)	MPF(Hz)	RMS(μV)	IEMG(μVs)
DS^*^	56.66 ± 5.53	68.14 ± 6.38	103.31 ± 44.54	8.02 ± 3.45	55.88 ± 5.17	65.57 ± 4.96^b^	85.02 ± 41.19^b^	6.72 ± 3.20^b^
	(54.60–58.72)	(65.76–70.52)	(86.68–119.94)	(6.73–9.30)	(53.95–58.72)	(62.72–66.42)	(69.64–100.40)	(5.53–7.92)
DS-HA^*^	57.21 ± 4.93	67.79 ± 5.83	113.00 ± 41.08	8.82 ± 3.15	56.51 ± 5.97	65.47 ± 6.08	105.81 ± 44.23^a^	8.30 ± 3.55^a^
	(55.37–59.05)	(65.61–69.96)	(97.66–128.34)	(7.65–9.99)	(54.27–58.74)	(63.20–67.74)	(89.30–122.33)	(6.98–9.63)
DS^**^	56.45 ± 5.99	68.30 ± 7.64	99.25 ± 31.14	7.83 ± 2.48	56.68 ± 6.45	66.53 ± 7.56	104.10 ± 48.75	8.27 ± 3.81
	(54.90–58.00)	(66.33–70.27)	(91.21–107.29)	(7.19–8.48)	(55.02–58.35)	(64.58– 68.49)	(91.51–116.70)	(7.25–9.25)
DS-HA^**^	56.58 ± 6.01	68.10 ± 7.94	103.18 ± 40.55	8.13 ± 3.25	56.50 ± 6.45	66.45 ± 7.92	109.50 ± 63.47	8.63 ± 4.97
	(55.03–58.14)	(66.05–70.15)	(92.71–113.66)	(7.29–8.97)	(54.83–58.17)	(64.40–68.50)	(93.10–125.90)	(7.35–9.92)

*DS* double-leg semisquat exercise, *DS-HA* double-leg semisquat exercise with hip adduction

*95 % CI* 95 % confidence intervals

^*^PFPS subjects ^**^healthy subjects

^a^Electric activity of the VMO muscles was significantly greater during DS-HA exercise than during DS exercise (*P* < 0.05)

^b^Electric activity of the VL muscle was significantly greater than that of the VMO muscle during DS exercise (*P* < 0.05)

## Discussion

This study shows that the values of RMS and IEMG of the VMO were significantly lower than the VL for PFPS patients. This indicates that the muscle fiber recruitment of VMO is weak when compared with VL. This supports the view that the muscle imbalance between the VMO and VL muscles exists in PFPS and could be one of the main factors leading to the development of PFPS [23]. The weakness of VMO could be examined by functional parameters, like: activation level, onset time or fatigue resistance, instead of structural parameters such as muscle length or fiber angle [24]. Surface EMG recordings provide a safe, easy, and noninvasive method that allows for objective quantification of functional parameters of the muscle.

The results from the present study revealed that within the CG there was no significant increase in either the activity of the VMO or VL when DS-HA was compared with DS. However, previous studies [16, 17, 25, 26] showed that an increase in the electrical activity of the quadriceps could be identified in healthy subjects when isometric hip adduction was combined with a knee flexion task. While reporting an increase in overall quadriceps activity, none of the previous studies demonstrated preferential recruitment of the VMO. This phenomenon might be explained by the good functional condition of the VMO in healthy subjects. The VMO can quickly accommodate the load changes of the patellofemoral joint. In other words, in healthy subjects, the motor unit recruitment of the VMO has reached a good state even without hip adduction, so the advantage of hip adduction is relatively less noticeable. Furthermore, some of those studies [16, 17] collected EMG data during dynamic contractions, and the analysis of EMG data of dynamic exercise can be very complicated since the EMG signal can be altered during the dynamic phase and the analysis of EMG data collected during dynamic exercise can be very complicated [27]. In general, we believe that compared with dynamic activity, the isometric contraction is preferred for the stability of EMG data.

With regards to PFPS patients, a consensus has not been reached regarding the activation of the VMO and VL muscles during semisquat exercises with and without hip adduction in individuals with PFPS. Our study demonstrated that within the PFPS group there was a significant increase only in the activity of the VMO muscles when DS-HA was compared with DS. However, Laprade, et al. [20] investigated the surface EMG activity of the VMO relative to the VL, during five isometric exercises in eight PFPS female subjects, and concluded that hip adduction, or the combination of hip adduction and knee extension, did not result in greater recruitment of the VMO as compared to the VL. In another study, Coqueiro, et al. [19] suggested that the association of hip adduction promoted a greater balance between the medial and lateral portions of the quadriceps femoris muscle, although there was no significant preferential activation of VMO over VL. The activity of the VMO and VL both increased at the same time; therefore the theory of preferential activation was not supported. The present study revealed that increased activity of EMG signals was only observed in the VMO, and not in the VMO and VL, when PFPS subject performed a semisquat with hip adduction. This finding indicates that a more selective activation of the VMO can be obtained in exercises that are combined with hip adduction. The main difference between our study and previous studies is that preferential activation of VMO in DS-HA were observed and promoted a greater balance of quadriceps. The conflicting results may be due to the differences in exercise execution between studies, such as the type of resistance applied to hip adduction or the degree of flexion of the knee and hip.

The preferential activation of VMO caused by hip adduction could be explained by the strong anatomical relation between the adductor muscles and the VMO muscle. Anatomic cadaver studies have shown that fibers from the VMO originate from the distal part of the adductor longus and magnus [28]. These hip adductors may give the VMO a stable origin when it contracts. With the addition of hip adduction there is a stretch to the VMO muscle, which would alter length tension properties, thus contributing to an enhanced contraction force. In other words, hip adduction can promote co-contraction of the VMO.

Double-leg semisquat, a closed kinetic chain exercise, may promote a more balanced activation of the quadriceps initially than an open kinetic chain exercise as seen in previous studies [29–32] and therefore lead to an improved subjective and clinical outcome in patients with anterior knee pain. Since closed kinetic chain exercise simulates many functional movements in daily activities, it may be better to incorporate task-related exercise into rehabilitation. Closed chain exercises are assumed to be a more functional intervention and have higher efficiency of the vastus medialis muscle than open chain exercises [30]. Furthermore, it has been suggested that closed kinetic chain exercises are safer because of a lower amount of shear force between the tibiofemoral joint surfaces in the functional range of motion [33]. In addition, open kinetic chain knee extension exercises may produce significantly greater activation of VL than closed kinetic chain exercises [13]. This may be of importance when designing training programs aimed at control of the patellofemoral joint.

The appropriate degree of knee flexion when performing the semisquat is another potentially controversial question. Earl et al. [17] reported that mini-squat exercise in combination with 45° knee flexion with hip adduction can significantly increase the activity of the quadriceps. However, Tang et al. [32] investigated the electromyographic activity of the quadriceps muscle in different knee angles. Maximal VMO/VL ratio was observed at 60° knee flexion, and muscle contraction intensity was also the greatest at this angle. This result indicated that a more selective activation of the VMO can be obtained at 60° knee flexion compared with other angles. Similar results were found in our preliminary experiments, and all subjects withstood the intensity of the action without worsening of symptoms or any other discomfort. Based on our data we support semisquat at 60° knee flexion as a desirable and safe exercise and EMG evaluation in patients with PFPS. Further study is needed to determine the effects of different knee flexion angles or load intensity on patients with PFPS.

The surface electromyography is a useful tool to evaluate the neuromuscular functional state and activity level. It assists in determining the correct protocol and, even more importantly, determining if the chosen exercises are really contributing to prompt and quick muscle recovery and to design the rehabilitation program individually. Although the noninvasive nature of surface EMG makes this technique ideal for clinical use and research, surface EMG data can be variable, which raises questions about the reliability of this technique. In this present study, ICC, which is considered to be a direct approximation of reliability, has been used for reliability assessment. Besides this, a standardized method for surface EMG electrode placement and isometric exercises are used in our study to establish the repeatability of EMG data.

The normalization of the raw EMG data is of primary concern if one intends to study and compare data collected from different muscles and different individuals in varying situations. As far as the data analysis, EMG ratio or percentage of the maximum voluntary contraction (MVC) is a common method to normalize EMG data. Since the MVC normalization approach probably results in an overestimate of the force produced, it is often suggested that comparing a subject to himself or herself can provide more precise data than comparison across individuals [34]. Posture, joint position, time, skin impedance and electrode placement were strictly standardized in the present study, along with strict inclusion and exclusion criteria to maximize external validity. It may be argued that the same hip adduction resistance was used for different subjects. However, within-subject analysis and paired t-tests were used in this study. In other words, comparison was performed only between the muscles within a particular subject and within a particular posture. Although the same resistance was used, this did not affect our judgment on the balance relationship between muscles. In addition, previous literature highlighted problems with failure to standardize adduction forces [19]. Therefore, interpretation of findings should be made with caution.

Although several studies have been done to analyze the electromyographic activity of the VMO and VL, most of them only use the time domain index (such as RMS), which reflects the level of physiological activity of the motor units and muscle contraction [13, 15–17, 19, 29, 32]. In the present study, the time domain index, both RMS and IEMG, revealed that VL had a higher level of activation than VMO and that hip adduction might promote motor unit recruitment of VMO for patients with PFPS. However, few investigations based on the frequency domain indexes have been performed to analyze the electromyographic fatigue characteristics of the quadriceps in PFPS [23]. To our knowledge, this is the first time that both the time domain index and the frequency domain index were analyzed to complete the comparison between VMO and VL, which may provide a more comprehensive understanding of the muscle function. Based on our data, we can conclude that VMO dysfunction is reflected not only in muscle contraction but also in fatigability.

Whereas, for the frequency domain indexes, which are derived from the spectral analysis and decrease with muscle fatigue [27], the statistically significant difference of VMO and VL was present only in MPF instead of both MPF and MF. This inconsistent response might be due to the different reliability and validity of MPF and MF at low muscle contraction levels. MPF is more sensitive for evaluation of muscle fatigue, especially under low load conditions [35]. In the present study, since the exercise load was relatively low and the test time for each exercise was only one minute, which might be too short to lead to severe fatigue, MPF was more likely to present the difference compared with MP. If the load and the time increased, more outcomes might be obtained for the frequency domain indexes. But at the same time, this could aggravate the symptoms of the patient. Based on MPF outcome in our study, the muscle endurance of VL is better than VMO for PFPS.

### Study limitations

There were some limitations of the study: 1) Static posture at 60° knee flexion was used for the study, but to determine the effects of different knee flexion angles or dynamic exercise, further study is needed; 2) Since patients with a VAS score greater than six were not enrolled in the current study, the results applied to patients with severe pain should be approached cautiously; 3) Since this work investigated only EMG data and did not use other tools, such as MRI, CT or ultrasonography – these could be added to investigate muscle imbalances in a future study.

## Conclusions

Based on our study, the weakness of the VMO, which leads to imbalance of the quadriceps, was apparent in the VMO of patient with PFPS. Furthermore, these findings indicate that preferential VMO activation can be obtained through DS-HA, which provides objective evidence to support the use of hip adduction during squat exercises in order to promote a better balance between VL and VMO, and to selectively strengthen the VMO in patients with PFPS. This study help to better understand the muscular mechanism, which will eventually help to guide and provide objective measurements in rehabilitation training and to design an exercise protocol for VMO strengthening. However, to confirm this statement, further prospective randomized controlled clinical trials would be necessary to compare the effects of different muscle training programs based on the semisquat with hip adduction exercise.

## Abbreviations

CG: Control group
DS: Double-leg semisquat
DS-HA: Double-leg Semisquat with hip adduction
IEMG: Integrated EMG
MF: Median frequency
MPF: Mean power frequency
PFPS: Patellofemoral pain syndrome
RMS: Root mean square
EMG: Electromyography
SG: Study group
VL: Vastus lateralis
VMO: Vastus medialis oblique
